# An efficient elliptic curve-based deterministic measurement matrix for micro-seismic data acquisition

**DOI:** 10.1371/journal.pone.0329793

**Published:** 2025-08-20

**Authors:** Haiqiang Liu, Guangjian Ge, Ze Jiang, Xiaojing Chen

**Affiliations:** 1 School of Computer Engineering, Jinling Institute of Technology, Nanjing, China; 2 CCTEG Changzhou Research Institute, Changzhou, China; 3 School of Computer and Electronic Information, Nanjing Normal University, Nanjing, China; Jaramogi Oginga Odinga University of Science and Technology, KENYA

## Abstract

Micro-seismic monitoring plays a critical role in geological disaster prediction. Current compressed sensing approaches for micro-seismic data acquisition, however, are hampered by measurement matrices with high computational complexity and inadequate reconstruction accuracy, particularly in resource-constrained sensor networks. To overcome these limitations, we propose an novel deterministic measurement matrix based on elliptic curve pseudo-random sequences. The proposed matrix offers three key advantages: (1) it requires only M×(N−1) addition operations. (2) it demonstrates lower mutual coherence than existing alternatives. And (3) experimental validation shows consistent performance advantages, achieving lower relative errors than Gaussian random, Chaotic, PEG, and Bernoulli matrices across both noise-free and noisy conditions. These combined improvements in computational efficiency, matrix incoherence, and reconstruction fidelity make our solution ideally suited for real-time micro-seismic monitoring in energy-limited field deployments.

## 1. Introduction

Micro-seismic monitoring technology [1] can monitor micro-seismic events caused by the inelastic deformations of rock masses in real time, provide early warning for the instability of rock masses, and effectively prevent disasters such as landslides, rock bursts, and fractures. It is widely used in geotechnical engineering applications such as mining [2], tunneling [3] and slope stability [4]. In the micro-seismic monitoring system, numerous micro-seismic sensors are deployed and self-organize into a Micro-Seismic Monitoring Wireless Sensor Network (MMWSN). The MMWSN holds great potential; however, it is constrained by limited energy and computational capabilities. According to traditional Nyquist sampling theory, the MMWSN needs to process and transmit a large amount of data, which poses a significant challenge. There is an urgent need to develop an efficient and simple data gathering method.

Compressed Sensing (CS) [5] is a novel information gathering theory that transcends the limitations of Nyquist sampling theory. Donaho et al. [5] have demonstrated that if a signal can be sparsely represented in a certain basis, it can be observed with a high compression ratio. Distributed Compressed Sensing (DCS) [6] extends CS to accommodate multiple signals. It leverages both intra-signal and inter-signal correlations to compress data, making it particularly well-suited for MMWSN. In DCS, signals are compressed by being projected onto a certain measurement matrix in the sensor and are jointly recovered at the central node.

Several studies have applied compressed sensing to micro-seismic data acquisition in MMWSNs. For instance, [7] proposed a systematic adaptive compressed sensing framework for micro-seismic monitoring, focusing on dictionary learning and adaptive recovery. Additionally, CS-based reconstruction advanced denoising techniques based on non-convex sparse optimization [8] have shown excellent performance in seismic signal processing. Segmentation-based compression methods integrating CS principles have been proposed in [9]. By tailoring the compression process to the signal sparsity characteristics and adopting enhanced recovery algorithms, these methods achieve higher accuracy and efficiency in signal transmission, contributing to extended network lifetime. However, most of these studies focus on signal recovery, while relatively few efforts have been made towards designing efficient measurement matrices for resource-constrained micro-seismic sensor networks.

There are mainly two types of measurement matrices for DCS: random measurement matrices and deterministic measurement matrices. The random measurement matrix, with elements drawn from a random Gaussian distribution [10] or a Bernoulli distribution [11], satisfies the Restricted Isometry Property (RIP) [11] with high probability. However, in practical applications, the random nature of the measurement matrix necessitates its transmission from the encoder to the decoder, thereby imposing additional communication burdens. Contrary to the random measurement matrix, the deterministic measurement matrix does not change with each sample. Therefore, it can be stored in the decoder in advance, making it more practical. Inspired by the inherent randomness and the strong incoherence properties of pseudo-random sequences, researchers have proposed several deterministic measurement matrices, such as the logistic chaotic sequence matrix [12], the tent chaotic sequence matrix [13], and the Chebyshev chaotic sequence matrix [14], among others. However, all these measurement matrices exhibit high computational complexity, making them unsuitable for the computationally constrained MMWSN. Dimakis et al. [15] investigate the relationship between channel coding and compressed sensing, proving that the check matrix of the low-density parity-check code can serve as a deterministic measurement matrix. Building on this theory, Lu et al. [16] propose a simple deterministic sparse measurement matrix using the Progressive Edge Growth (PEG) method, which consists solely of two elements, “0” and “1”. However, its performance still requires improvement.

In this paper, we propose a deterministic binary measurement matrix based on the elliptic curve sequence [17], which we refer to as the elliptic curve measurement matrix. Simulation results show that the elliptic curve measurement matrix exhibits lower mutual coherence and outperforms several state-of-the-art measurement matrices in MMWSN data gathering. Additionally, because the elliptic curve measurement matrix consists solely of two elements, ‘0’ and ‘1’, and requires only a minimal number of additions in the sensor, it has low computational complexity. This characteristic makes it especially suitable for MMWSNs with limited computational capacity. This paper is organized as follows: Section II reviews the MMWSN, the theory of DCS, and the theory of elliptic curve sequence. In Section III, the method for constructing a measurement matrix based on the elliptic curve sequence is proposed, and its characteristics are analyzed. In Section IV, the performance of the proposed measurement matrix is demonstrated using real micro-seismic data. Section V presents the conclusion.

## 2. Preliminaries

### 2.1. MMWSN

Generally, micro-seismic events can be understood as earthquakes with short duration, small influence range, and less energy release. Micro-seismic monitoring technology is a three-dimensional spatial monitoring technology for the micro-fracturing of rocks. It can effectively monitor the occurrence time, spatial position, and energy of the fracture point in the rock mass by utilizing the characteristics of micro-seismic signals, thereby predicting the deformation and damage of the rock.

Most existing micro-seismic monitoring systems are based on wired communications, which suffer from inflexibility, high complexity in expansion, and limited monitoring range. The micro-seismic monitoring system based on wireless sensor networks can effectively resolve these issues [18]. A large number of micro-seismic sensors are deployed in the rock and self-organize into an MMWSN, which offers the advantages of low cost, easy expansion, and high reliability. MMWSN can achieve comprehensive monitoring coverage of disasters and effectively enhance the level of geological disaster forecasting.

**Fig 1:**
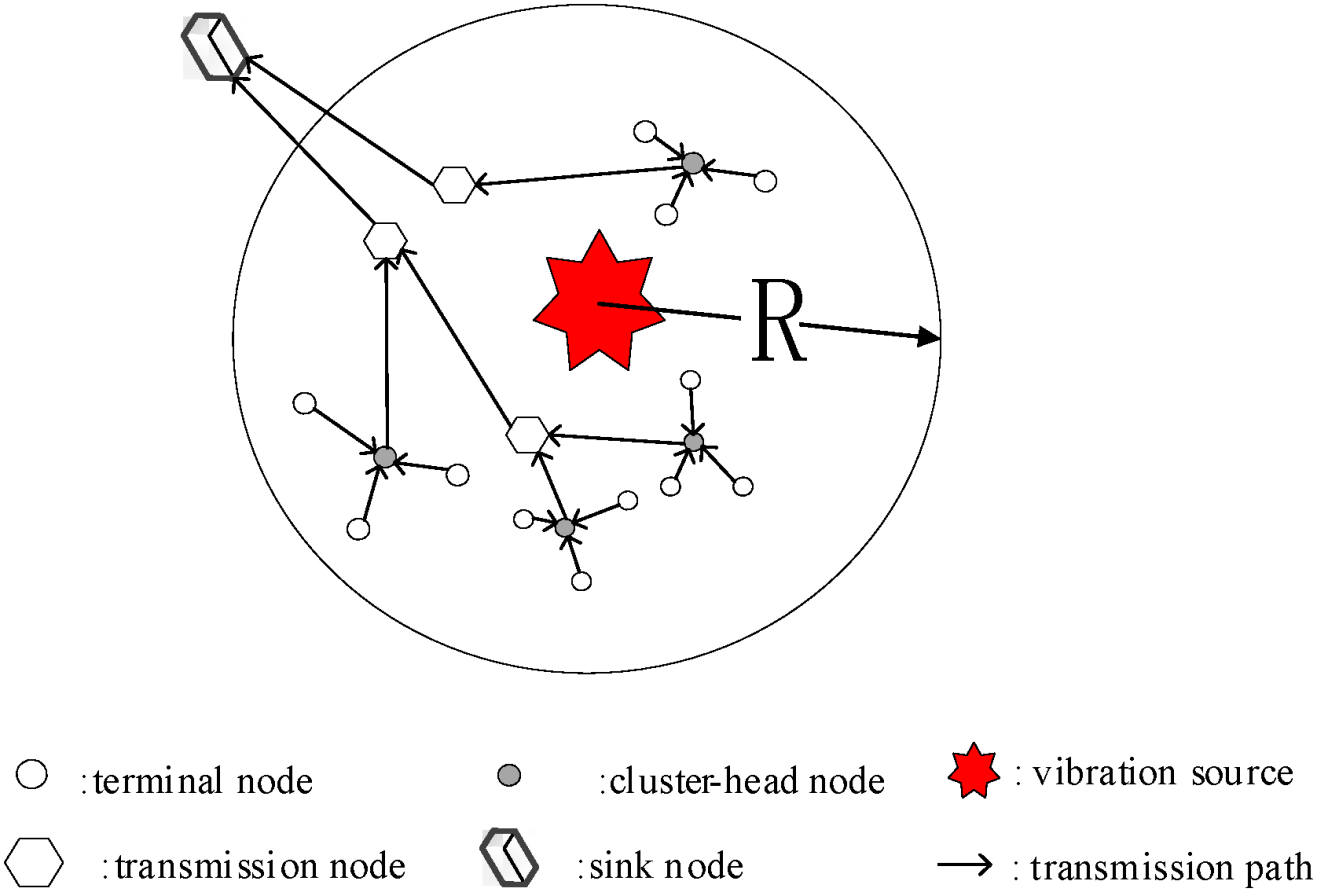
Diagram of MMWSN.

The network structure of the MMWSN is shown in Fig 1. In a three-dimensional area, a large amount of sensors are arranged in uniform, random or optimal distribution. MMWSN is composed of terminal nodes, cluster-head nodes, transmission nodes, and sink nodes. Terminal nodes sense the environment and upload data to the cluster-head nodes. Cluster-head nodes aggregate the received data and transmit it to the transmission nodes. Transmission nodes simply relay data. After one or more hops, the data reach the sink node, which then uploads the data to the server for processing.

Traditionally, micro-seismic signals are sampled in the terminal node according to the Nyquist sampling theorem before being transmitted to the cluster-head node. However, data gathering methods based on the Nyquist sampling theorem require a very high sampling frequency, substantial computational complexity, and significant storage space, making them unsuitable for resource-constrained MMWSNs. There is an urgent need to develop simple and efficient data gathering methods for MMWSNs. DCS represents one of the best options.

### 2.2. Theory of DCS

DCS is an extension of CS. Before introducing the theory of DCS, let us first discuss the theory of CS.

*1) Compressed sensing:* CS is proposed by Donaho et al. [5] It breaks the limitations of the Nyquist sampling theorem. Donaho et al. [5] proved that the information from sparse signals can be obtained with a very low sampling rate, and after being transmitted to the server, they can be perfectly reconstructed by solving an optimization problem.

The prerequisite for applying CS to compress data is that the signal must be sparse. If signal $𝐱\in {𝐑}^{N}$ can be represented as 𝐱=Ψθ and there are only k≪N non-zero elements in θ, then 𝐱 is called a sparse signal and Ψ is referred to as the sparse basis.

Researchers have proposed several methods for sparse representation, including the Discrete Fourier Transform (DFT) [19], wavelet transform [20], over-complete dictionaries [21] and dictionary learning [19]. In this paper, we use the Discrete Fourier Transform (DFT) to represent micro-seismic signals. The sparse basis based on DFT is defined as (1).


$$\Psi =[\begin{array}{cccc} W_{N}^{\left(0)(0\right)} & W_{N}^{\left(0)(1\right)} & \cdots & W_{N}^{\left(0)(N-1\right)}\\ W_{N}^{\left(1)(0\right)} & W_{N}^{\left(1)(1\right)} & \cdots & W_{N}^{\left(1)(N-1\right)}\\ \vdots & \vdots & \ddots & \vdots \\ W_{N}^{\left(N-1)(0\right)} & W_{N}^{\left(N-1)(1\right)} & \cdots & W_{N}^{\left(N-1)(N-1\right)} \end{array}]$$ (1)

where $W_{N}^{\left(a)(d\right)}=e^{-j\frac{2\pi }{N}ad},0\leq a\leq N-1,0\leq d\leq N-1$.

**Fig 2:**
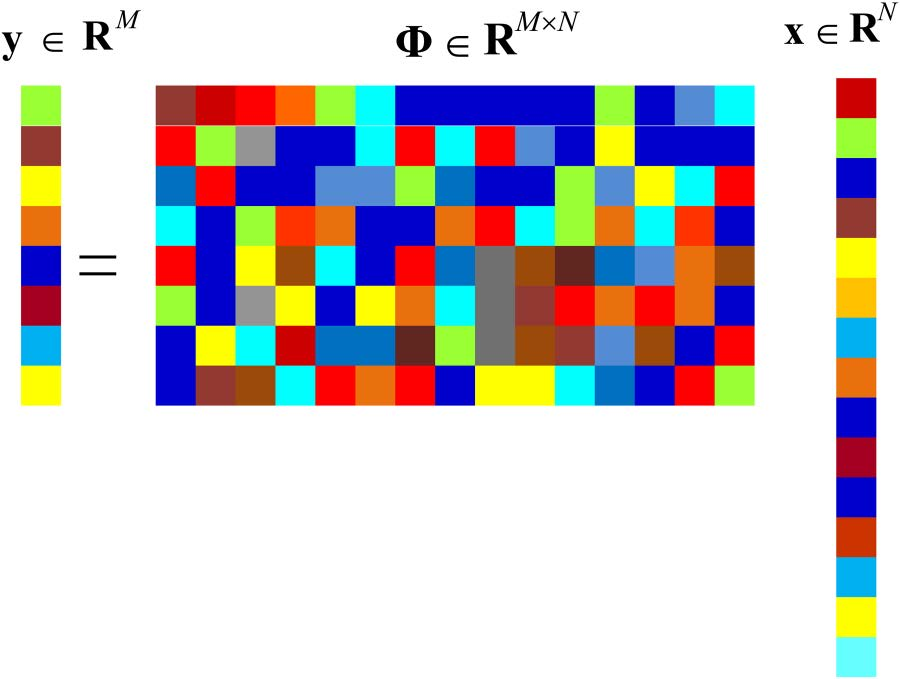
Nonlinear projection in CS.


According to Compressed Sensing (CS) theory, a sparse signal 𝐱 can be compressed by a nonlinear projection, as shown in Fig 2:

𝐲=Φ𝐱 (2)

where $\Phi \in {𝐑}^{M*N}(M<N)$ represents the measurement matrix.

Clearly, the *N*-dimensional signal 𝐱 is compressed into an *M*-dimensional signal 𝐲. Theoretically, it is unsolvable to recover 𝐱 from 𝐲. However, Donaho et al. [5] prove that if the sparsity of 𝐱 is k, and the measurement matrix Φ satisfies the Restricted Isometry Property (RIP) of order 3k with $\delta_{3k}\in (0,1)$:


$$\left(1-\delta_{3k}\right){\left\|𝐱\right\|}_{2}^{2}\leq \left\|\Phi_{w}𝐱\right\|\leq \left(1+\delta_{3k}\right){\left\|𝐱\right\|}_{2}^{2}$$ (3)

where w⊂{1,2,⋯,N}, |w|≤3k, 𝐱 can be perfectly recovered by solving the optimization problem as shown in (4).


$$\min{\left\|\Psi^{T}x\right\|}_{1}s.t.𝐲=\Phi 𝐱$$ (4)

Computing the the RIP [11] of a measurement matrix is challenging. Research indicates that the mutual coherence of a sensing matrix can be used to evaluate its performance [22]. The lower the mutual coherence of a sensing matrix, the greater its data compression capability. The mutual coherence of the sensing matrix 𝐀 is defined as Equation (5).


$$\mu \left(𝐀\right)≜\max_{i\neq j}\frac{\left|\left\langle a_{i},a_{j}\right\rangle \right|}{{\left\|a_{i}\right\|}_{2}{\left\|a_{j}\right\|}_{2}}1\leq i,j\leq N$$ (5)

where $a_{i}$ is the i−th column of 𝐀.

**Fig 3:**
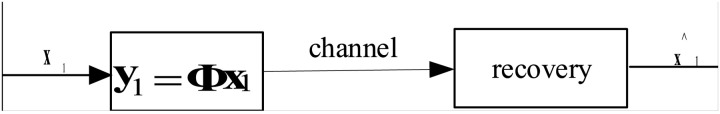
Structure of CS.

The structure of CS is illustrated in Fig 3. Evidently, CS only utilizes intra-signal correlation for data compression, making it inefficient for data gathering in MMWSNs.

**Fig 4:**
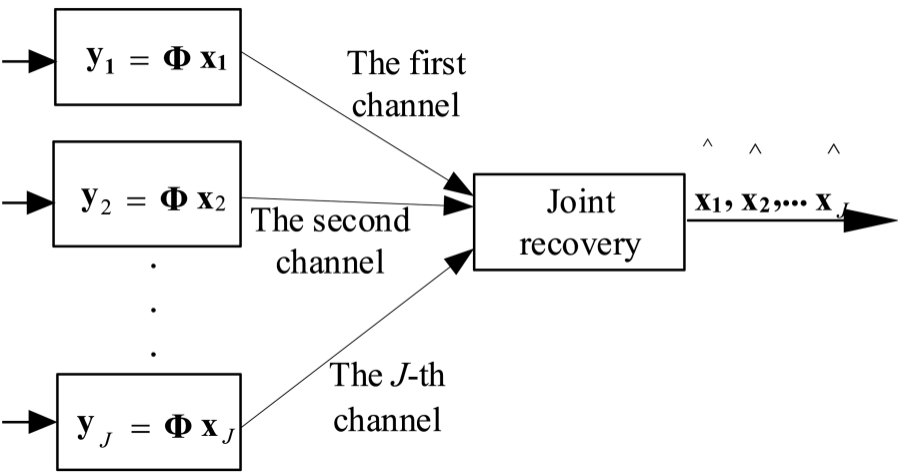
Structure of DCS.

*2) Distributed Compressed sensing:* DCS is an extended version of CS for WSN. It can utilize not only the intra-signal correlation but also the inter-signal correlation to compress data. The structure of DCS is illustrated in Fig 4. In sensors, signals are compressed through separate nonlinear projections and, once transmitted to the server, are jointly recovered by utilizing both intra-signal and inter-signal correlations. Obviously, DCS is more suitable for MMWSN.

A prerequisite for applying DCS to data compression is that the signals exhibit joint sparsity. J signals ${𝐱}_{j}(j=1,2,\cdots ,J)$ constitute a joint signal $𝐗=\left[{𝐱}_{1},{𝐱}_{2},\cdots ,{𝐱}_{J}\right]$. According to the joint sparse theory, the joint signal 𝐗 can be represented as


$$𝐗=\Psi \Theta =\Psi [\theta_{1},\theta_{2},\cdots ,\theta_{J}\backslash ]$$ (6)

We define ℜ(Θ\)as the joint support set, which comprises the indices of the non-zero rows of Θ’s non-zero rows. Furthmore, K=|ℜ(Θ)| is defined as the joint sparsity.

The nonlinear projections of J signals can be represented as


$$𝐘=\Phi 𝐗=\Phi \left[{𝐱}_{1},{𝐱}_{2},\cdots ,{𝐱}_{J}\right]$$ (7)

where 𝐘 is called the jointly compressed signal. The theory of DCS demonstrates that if 𝐗 is joint sparse, it can be recovered by solving the optimization problem presented in Equation (8).


$$\begin{array}{c} \min_{\Theta }\left|ℜ\left(\Theta \right)\right|,\text{s.t.}𝐘=𝐀\Theta \end{array}$$ (8)

Once Θ is recovered, 𝐗 can be restored through an inverse sparse transformation.

### 2.3. Theory of elliptic curve sequences

If a sequence has a predetermined structure, can be repeatedly generated, and possesses random characteristics, it is referred to as a pseudo-random sequence [23]. Because a pseudo-random sequence exhibits good randomness, it is widely used in spread-spectrum communication [24], multiple-access communication [25] data encryption [26], and more. There are mainly two types of pseudo-random sequences: one is based on mathematical theory, such as the elliptic curve sequence [27], the linear congruence sequence [28] and the Gordon-Mills-Welch sequence [29]; the other is based on linear feedback shift registers, such as the filter sequence [30].

Let ξ be an elliptic curve over a finite field ${𝐅}_{q}$ where $q=p^{m}$, with p being a prime number and m a positive integer. For q>3, the elliptic curve ξ is defined by the following equation:


$$y^{2}=x^{3}+ax+b(modq),a,b\in {𝐅}_{q}$$ (9)

where $4a^{3}+27b^{2}(modq)\neq 0$.

Denote ξ(${𝐅}_{q}$) as the set consisting of all the rational points on ξ and a point at infinity. Let t represent the cardinality of the set ξ(${𝐅}_{q}$), which is referred to as the order of the elliptic curve ξ. According to the Hasse’s theorem [31], t is very close to q, and it is always possible to find an elliptic curve with order ($q-1-2q^{1/2})\leq t\leq (q-1+2q^{1/2})$.

Researchers have proposed many fast algorithms for computing the order of elliptic curves, such as the SEA (Schoof-Elkies-Atkin) [32] algorithm, Schoof’s algorithm [33] and the satoh algorithm [34].

The binary elliptic curve sequence can be constructed using the trace function [35], the discrete logarithm [31] and the Legendre symbol [17]. L. Goubin et al. [17] constructed five types of binary pseudo-random sequences using the Legendre symbol and the polynomial functions. Let $G=\left(u_{l},v_{l}\right)\in {𝐅}_{p}\times {𝐅}_{p}$ be the l−th rational point on ξ where 1≤l≤t−1; then five types of binary sequences $S=\left\{s_{1},s_{2},\cdots ,s_{t-1}\right\}$ can be constructed as follows.


$${ConstructionI:s}_{l}=\left\{ \begin{array}{c} 1,v_{l}>\frac{p}{2}\\ 0,else \end{array}\right.$$ (10)

$$ConstructionII:s_{l}=\left\{ \begin{array}{c} 1,u_{l}>\frac{p}{2}\\ 0,else \end{array}\right.$$ (11)

$$ConstructionIII:s_{l}=\left\{ \;\;\;\;\begin{array}{c} 1,\left(v_{l}mod2=0\right)\\ 0,else \end{array}\right.$$ (12)

$$ConstructionIV:s_{l}=\left\{ \;\;\;\;\begin{array}{c} 1,\left(u_{l}mod2=0\right)\\ 0,else \end{array}\right.$$ (13)

$$ConstructionV:s_{l}=\left\{ \;\;\;\;\begin{array}{c} 1,\left(u_{l}<v_{l}\right)\\ 0,else \end{array}\right.$$ (14)

These five types of elliptic curve sequences have been proven to possess good auto-correlation and cross-correlation properties. We can always construct a binary sequence of a specified length of t−1 using equation (10)–(14). In this paper, we utilize these five types of elliptic curve sequences to construct deterministic binary measurement matrices.

## 3. Deterministic bipolar measurement matrix based on elliptic curve sequence

### 3.1. Construction of the elliptic curve measurement matrix

We construct an M×N deterministic bipolar measurement matrix using an elliptic curve in four steps.

1. Choose q,aandb that satisfy ($q-1-2q^{1/2})\leq N+1\leq (q-1+2q^{1/2})$ and $4a^{3}+27b^{2}(modq)\neq 0$.
2. For each triple (q,a,b), compute the order of the elliptic curve using the SEA (Schoof-Elkies-Atkin) algorithm [32], and select those elliptic curves with an order of N+1.
3. Construct the elliptic curve sequence $S=\left\{s_{1},s_{2},\cdots ,s_{N}\right\}$ by using equation (10) through (14).
4. Define a mapping f(z)=1−2z, and construct a temporal matrix 𝐐 using equation (15).
5. Construct the elliptic curve measurement matrix by selecting the first M,(1<M<N) rows from 𝐐.

$$Q=[\begin{array}{ccccc} {f(s}_{1}) & {f(s}_{2}) & \cdots & {f(s}_{N-1}) & f(s_{N})\\ {f(s}_{2}) & {f(s}_{3}) & \cdots & f(s_{N}) & f(s_{1})\\ \vdots & \vdots & \ddots & \vdots & \vdots \\ {f(s}_{N-1}) & {f(s}_{N}) & \cdots & {f(s}_{N-3}) & f(s_{N-2})\\ f(s_{N}) & {f(s}_{0}) & \cdots & {f(s}_{N-2}) & f(s_{N-1}) \end{array}]$$ (15)

### 3.2. Analysis of RIP

As a pseudo-random sequence, an elliptic curve sequence possesses a 0–1 balance property. Consequently, the elements of the elliptic curve measurement matrix follow the distribution described in equation (16). As proven in [36,37], if the measurement matrix Φ adheres to the distribution specified in (16), it satisfies the RIP with high probability.


$$\phi_{i,j}=\left\{ \begin{array}{c} 1,p=\frac{1}{2}\\ -1,p=\frac{1}{2} \end{array}\right.$$ (16)

where $\phi_{i,j}$ is the element at the i−th row and j−th column of Φ. The relationship between the distribution of random symmetric signs and the Restricted Isometry Property (RIP) is summarized as follows.

**Lemma 1.** Let Φ be a random M×N matrix whose entries are drawn from a distribution with random symmetric signs. Given M, N, δ∈(0,1) and any $k\leq c_{0}M/log(N/k)$, there exists constants $c_{0},c_{1}>0$ that depend only on δ, as prescribed by $\delta_{3k}$ in equation (3), and Φ satisfies the RIP with probability greater than


$$1-2exp\left(-c_{1}M\right)$$

where


$$c_{1}\leq \frac{\left(3\delta^{2}-\delta^{3}\right)}{48-c_{0}\left[1+\left(1+\log(12/\delta )\right)/\log(N/k)\right]}$$

### 3.3. Analysis of mutual coherence

As described in Section II, the lower the mutual coherence of a measurement matrix, the greater its capability to acquire information. In this paper, the DFT basis is utilized as the sparse basis, and the mutual coherence of the elliptic curve measurement matrix is compared with those of the Gaussian random measurement matrix [10], the random sparse measurement matrix [38] and the chaotic measurement matrix [12].

**Fig 5:**
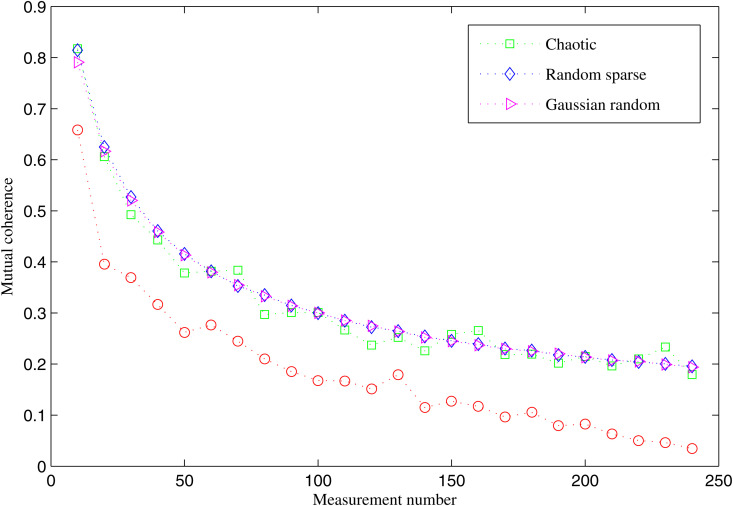
Mutual coherence of different measurement matrices.

With N fixed, we compare the mutual coherence of different measurement matrices according to the changes in the number of measurements, *M*. For example, an elliptic curve measurement matrix with N=255 is constructed using a=1,b=2andp=257. As shown in Fig 5, the elliptic curve matrix consistently achieves lower mutual coherence than the Gaussian random matrix, the random sparse matrix, and the chaotic matrix across different values of M. This indicates a better incoherence, which directly translates to improved reconstruction performance.

To evaluate robustness, we analyze the mutual coherence of 200 elliptic curve measurement matrices, constructed using random parameters a,b and q, where q is less than 1000. The numerical experiment results show that, across all measurement numbers changing from 10 to *N-*5, elliptic curve measurement matrices exhibit lower mutual coherence compared to the Gaussian random matrix, the random sparse matrix, and the chaotic matrix. These results confirm that the proposed elliptic curve matrix offers stronger incoherence compared to other measurement matrices. This makes it more effective for compressed sensing in practical MMWSN scenarios.

### 3.4. Analysis of computational complexity

The computational complexities of different measurement matrices are shown in Table 1. The Gaussian random measurement matrix and the chaotic measurement matrix are dense, meaning that every element requires multiplication and addition during the measurement process. They exhibit the highest computational cost, making them unsuitable for resource-constrained environments such as MMWSNs.

**Table 1:** Computational complexities of various measurement matrices.

Φ	Number of Multiplication Operations	Number of Addition Operations
**Gaussian random measurement matrix**	M×N	M×(N−1)
**Random sparse measurement matrix**	0	4×(N−1)
**Chaotic measurement matrix**	M×N	M×(N−1)
**GMW measurement matrix**	0	M×(N−1)
**PEG measurement matrix**	0	4×(N−1)
**Hadamard measurement matrix**	0	M×(N−1)
**Elliptic curve measurement matrix**	0	M×(N−1)

Other measurement matrices are either binary, containing only “0” and “1”, or bipolar, containing only “-1” and “1”. These matrices avoid multiplication entirely, relying solely on addition operations. Although the computational complexities of the PEG and random sparse measurement matrices are slightly lower than that of the elliptic curve measurement matrix, the difference in addition operations is negligible in practice.

More importantly, unlike the PEG matrix which requires a dedicated graph-based construction process, our elliptic curve matrix is generated using a deterministic mathematical sequence with low overhead, further reducing memory and implementation complexity.

From a computational standpoint, multiplication-free binary operations makes the proposed matrix a practical and energy-efficient choice, especially in embedded platforms or battery-powered wireless sensors.

## 4. Experimental result and analysis

In this section, we compare the performance of the elliptic curve measurement matrix with that of several state-of-the-art measurement matrices in the context of real coal mine micro-seismic data gathering. These matrices include the Gaussian random measurement matrix [10], the chaotic measurement matrix [12], the Bernoulli measurement matrix [11] and the PEG measurement matrix [16]. The experimental data are gathered using three ADXL362 3-axis MEMS accelerometer sensors, deployed on the coal mine working surface roof to monitor micro-seismic events. Let ${𝐱}_{1},{𝐱}_{2}$, and ${𝐱}_{3}$ be three micro-seismic signals gathered by these sensors during a micro-seismic event; the joint micro-seismic signal can be represented as $𝐗=\left[{𝐱}_{1},{𝐱}_{2},{𝐱}_{3}\right]$. The DFT sparse basis is used to sparsely represent the micro-seismic signals. In our simulations, ${𝐱}_{1},{𝐱}_{2},$ and ${𝐱}_{3}$ are compressively sampled using the measurement matrix and jointly recovered using the Simultaneous Orthogonal Matching Pursuit (SOMP) algorithm [6]. The average relative error from 100 trials is used to evaluate the performance of different measurement matrices. Relative error is defined as


$$Relativeerror=\frac{\left\|𝐗-\hat{𝐗}\right\|}{\left\|𝐗\right\|}$$ (17)

where 𝐗 is the joint micro-seismic signal and $\hat{𝐗}$ is the recovered joint micro-seismic signal.

### 4.1. Noiseless condition

**Fig 6:**
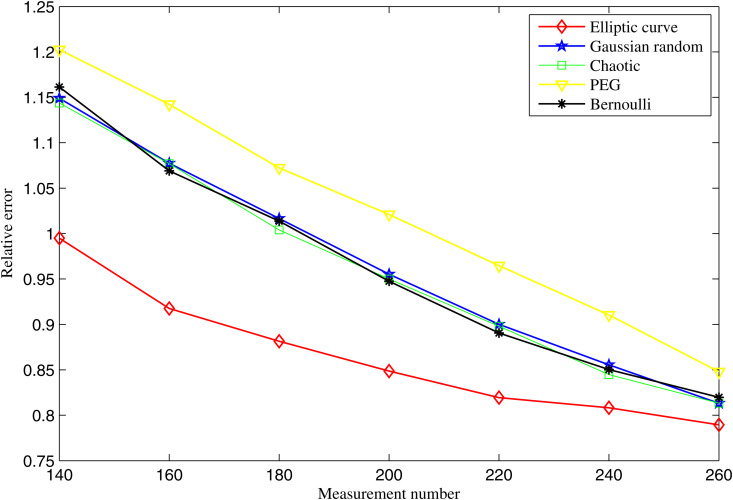
Average relative errors of different measurement matrices as a function of *M* in noiseless conditions.

In noiseless conditions, with N=540 and K=100, the variations in the average relative errors for different measurement matrices as a function of the number of measurements are shown in Fig 6. The average relative errors of all measurement matrices decrease as the number of measurements, M, increases. This is because a larger number of measurements allows the measurement matrix to capture more seismic information from the microseismic events.

The results clearly show that the average relative error of the elliptic curve measurement matrix is consistently lower than that of other measurement matrices. This trend indicates that the elliptic curve matrix is more efficient in utilizing the available measurements, leading to more accurate results with fewer measurements.

For example, when the number of measurements is set to M=180, the average relative error of our matrix is 0.882, compared to 1.075 for the PEG matrix, 1.012 for the Chaotic matrix, 1.020 for the Gaussian random matrix, and 1.021 for the Bernoulli matrix.

Moreover, due to its sequence-based construction, the elliptic curve matrix requires fewer computations, making it more suitable for real-time implementation in MMWSNs. These advantages demonstrate the superiority of our approach in both reconstruction accuracy and practical feasibility.

### 4.2. Noisy condition

Seismic data collection is often affected by noise, which can arise from various sources such as sensor interference, environmental disturbances, or background vibrations. In these noisy conditions, the micro-seismic data acquisition process can be represented as


𝐘=Φ𝐗+noise (18)

where noise is Gaussian white noise. The noise intensity can be measured with Signal Noise Ratio (*SNR*), which is defined as


$$SNR=\frac{\sigma_{𝐘}^{2}}{\sigma_{noise}^{2}}$$ (19)

where $\sigma_{𝐘}^{2}$ is the variance of the jointly compressed signal, and $\sigma_{noise}^{2}$ is the variance of the noise signal.

**Fig 7:**
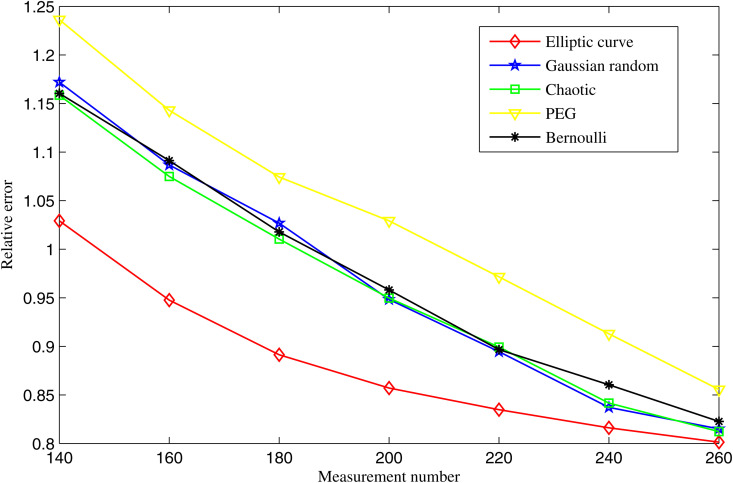
Average relative errors of different measurement matrices as a function of *M* with *SNR* = 20db.

With N=540, K=100 and SNR=20, the variations in the average relative errors for different measurement matrices as a function of the number of measurements are illustrated in Fig 7. Although the average relative errors of all methods decrease with increasing number of measurements, our proposed matrix consistently achieves the lowest relative error.

For example, when M=200, the relative error of our method is 0.855, while the PEG method shows 1.041, the Chaotic matrix shows 0.951, the Gaussian random matrix shows 0.952 and the Bernoulli matrix shows 0.959.

This clearly demonstrates the superior stability and robustness of the elliptic curve measurement matrix when dealing with noisy seismic data. The strong pseudo-random structure of the matrix help it efficiently capture signal features while being less sensitive to measurement noise.

**Fig 8:**
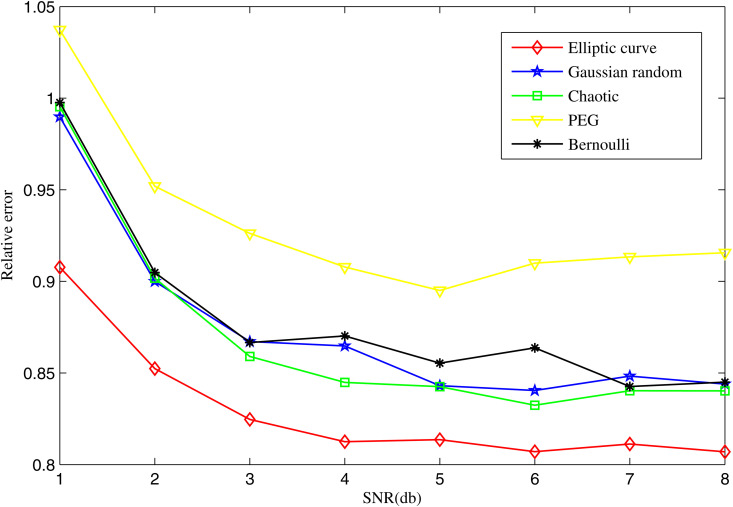
Average relative errors of different measurement matrices as a function of *SNR* with *M* = 240.

Fig 8 shows the average relative errors of different measurement matrices as a function of SNR changes with N=540, K=100 and M=240. As the SNR decreases, all methods suffer performance degradation, but our matrix maintains a consistently lower error curve across all SNR levels.

This implies a higher resilience to noise compared to other measurement matrices. From a practical standpoint, this robust noise tolerance means that fewer measurements are required to achieve the same level of accuracy, enabling significant savings in energy consumption, memory usage, and communication cost-all critical concerns in MMWSN deployments.

These results confirm that the elliptic curve measurement matrix not only surpasses traditional measurement matrices in noiseless environments but also maintains superior robustness and reconstruction performance in noisy conditions, making it a highly promising choice for real-world micro-seismic monitoring applications.

## 5. Conclusions

A simple and efficient deterministic measurement matrix based on the elliptic curve pseudo-random sequence for micro-seismic data gathering is proposed. Initially, the theories of Micro-Seismic Monitoring Wireless Sensor Networks (MMWSN), DCS, and elliptic curve sequences are introduced. Then, the method for constructing the deterministic binary measurement matrix based on the elliptic curve sequence is presented. Furthermore, it is demonstrated that the elliptic curve measurement matrix possesses low mutual coherence and low computational complexity, which aligns with the MMWSN’s characteristic of limited resources. Simulations on real micro-seismic data verify that the proposed measurement matrix outperforms some state-of-the-art measurement matrices under noiseless and noisy conditions.

Despite these promising results, our method still has some limitations. First, due to the intrinsic constraints of elliptic curve pseudo-random sequence generation, the number of columns in the measurement matrix cannot be arbitrarily large, which limits its direct applicability to signals of arbitrary length. Second, although the method shows excellent performance in the presence of Gaussian noise, further testing is needed under more complex and dynamic communication conditions. These limitations will be addressed in our future work.
